# High-pressure X-ray diffraction, Raman, and computational studies of MgCl_2_ up to 1 Mbar: Extensive pressure stability of the *β*-MgCl_2_ layered structure

**DOI:** 10.1038/srep30631

**Published:** 2016-08-12

**Authors:** Elissaios Stavrou, Yansun Yao, Joseph M. Zaug, Sorin Bastea, Bora Kalkan, Zuzana Konôpková, Martin Kunz

**Affiliations:** 1 Lawrence Livermore National Laboratory, Physical and Life Sciences Directorate, P.O. Box 808 L-350, Livermore, California 94550, USA; 2 Department of Physics and Engineering Physics, University of Saskatchewan, Saskatoon Saskatchewan, S7N 5E2, Canada; 3 Canadian Light Source, Saskatoon, Saskatchewan, S7N 2V3, Canada; 4 Advanced Light Source, Lawrence Berkeley Laboratory, Berkeley, California 94720, United States; 5 Advanced Materials Research Laboratory, Department of Physics Engineering, Hacettepe University 06800, Beytepe, Ankara, Turkey; 6 DESY Photon Science, D-22607 Hamburg, Germany

## Abstract

Magnesium chloride (MgCl_2_) with the rhombohedral layered CdCl_2_-type structure (*α*-MgCl_2_) has been studied experimentally using synchrotron angle-dispersive powder x-ray diffraction and Raman spectroscopy using a diamond-anvil cell up to 100 GPa at room temperature and theoretically using first-principles density functional calculations. The results reveal a pressure-induced second-order structural phase transition to a hexagonal layered CdI_2_-type structure (*β*-MgCl_2_) at 0.7 GPa: the stacking sequence of the Cl anions are altered resulting in a reduction of the c-axis length. Theoretical calculations confirm this phase transition sequence and the calculated transition pressure is in excellent agreement with the experiment. Lattice dynamics calculations also reproduce the experimental Raman spectra measured for the ambient and high-pressure phase. According to our experimental results MgCl_2_ remains in a 2D layered phase up to 100 GPa and further, the 6-fold coordination of Mg cations is retained. Theoretical calculations of relative enthalpy suggest that this extensive pressure stability is due to a low enthalpy of the layered structure ruling out kinetic barrier effects. This observation is unusual, as it contradicts with the general structural behavior of highly compressed AB_2_ compounds.

According to Pauling’s first rule, the ambient pressure crystal structure of divalent metal halides and oxides AX_2_ is mainly determined by the cation-anion radius ratio R = *r*_*c*_/*r*_*a*_^1^,^2^. For R values > 1, 9-fold coordinated cations ionic structures like cotunnite (*α*-PbCl_2_-type) are formed while for R > 0.73, 8-fold coordinated cations ionic structures like fluorite (CaF_2_ and SrCl_2_) are formed^3^. In the case of 0.73 > R > 0.41 the well-known rutile-type structure (e.g. TiO_2_ or MnF_2_) with 6-fold coordinated cations is mainly adopted^4^. For 0.35 < R < 0.41 cations remain in 6-fold coordination; however, 2D layered, in contrast to 3D fluorite and rutile, Lawrencite-type structures are favored like the CdCl_2_-type structure^5^. Thus, these layered structures hold a very interesting position in the AX_2_ compound phase diagram as intermediate between 3D structures with 4-fold coordinated cations for 0.35 < R (quartz-SiO_2_) and rutile-type (stishovite-SiO_2_). Lawrencite-type structures are characterized by edge sharing AX_6_ octahedra forming a “ sandwich” of X-A-X layers (Fig. 1) where the stacking of halide atoms may be cubic-close packed ABCABC along the c-axis (e.g. rhombohedral CdCl_2_-type^6^) or hexagonal close-packed ABABAB along the c-axis (e.g. hexagonal CdI_2_-type^7^,^8^) or consist of mixed stacking faults with a random succession of hexagonal and cubic close-packed layers^9^. In contrast to the fluorite or rutile type metal halides and oxides, studies of the structural behavior of compressed Lawrencite-type compounds appear to be very limited and to the best of our knowledge only FeCl_2_^10^,^11^ has been systematically studied by X-ray diffraction at pressures up to 65 GPa. At ambient conditions, MgCl_2_ with a R = 0.40^12^ crystalizes into alternative crystalline polymorphs, which is a common aspect of this class of inorganic X-A-X layered compounds: (a) the most common is *α*-MgCl_2_, CdCl_2_-type SG *R-3m* (166) Z = 3^5^,^13^,^14^ followed by the less common (b) *β*-MgCl_2_, CdI_2_-type SG *P-3m1* (164) Z = 1^9^. The computed density-functional theory energy difference between these phases is on the order of vibrational contributions to the structure (e.g., only 0.02 eV per formula unit volume)^2^. Mechanically milling MgCl_2_ can easily shift the polymorphic concentration ratio. The latter effect is industrially important given the role of MgCl_2_ to support titanium halide propagation centers (nucleation sites), prepared by milling, in Ziegler-Natta catalysts used to accelerate the polymerization of olefins^15^. To the best of our knowledge, no high pressure experimental structural study has been reported on MgCl_2_, plausibly because of its extremely high hygroscopicity. A previous theoretical study^2^ suggests that pressurized MgCl_2_ (*α* or *β* phases) transforms to the rutile structure at ≈17 GPa and the fluorite structure at ≈77 GPa *i.e.* trending towards higher connectivity or 3D-like structures as normally would be expected for AX_2_ inorganic compounds.

There are technological interests that motivate the characterization of extreme condition properties of MgCl_2_. It is well-known that a number of metals, metal oxides, and halide materials have extraordinarily effective antimicrobial properties. Halogen, interhalogen, and halogen oxide gases oxidize and disrupt the action of bacterial cells: membrane function related genes are repressed, primary metabolism related genes and protein synthesis genes are repressed, and amino acid synthesis genes become activated. Detonation chemistry may generate halide species and physical conditions that are especially effective at destroying/neutralizing bio-agents; however, large-scale tests can be relatively time consuming and expensive. Laboratory-scale measurements generate data required to semi-empirically calculate detonation performance including products^16^. Confident semi-empirical thermochemical calculations of chemistry under extreme pressure-temperature conditions are made using equations of state data and phase diagrams of formulated reactants and likely high concentration product materials. Magnesium (Mg) is an excellent candidate for use as an explosive additive due to its high heat of combustion and relatively low boiling point temperature^17^,^18^,^19^,^20^. In addition, MgO and halogen adducts show significant biocidal activity^21^. The reaction of Mg with chlorine (Cl) leading to MgCl_2_ production may occur for example in Mg-loaded energetic formulations with significant Cl content, e.g. due to the use of an oxidizer such as ammonium perchlorate (AP) e.g. ref. 22. The use of MgCl_2_ as an additive in energetic formulations is also a plausible route to achieving high biocidal activity. In both scenarios, knowledge of the MgCl_2_ equation of state (EOS) is crucial toward understanding and computing the reactive shock behavior of energetic systems that involve a significant amount of MgCl_2_.

In order to examine the high pressure structural behavior of MgCl_2_ and also to expand knowledge of the high pressure structural behavior of layered-structured metal halide compounds we have carried out a detailed synchrotron angle-dispersive powder x-ray diffraction and Raman spectroscopy studies up to 100 GPa and compared these results with first-principles density functional calculations. We report a second-order phase transition to the CdI_2_-type structure at ≈0.7 GPa that remains stable up to 1 Mbar. Remarkably, the high pressure MgCl_2_ phase retains a 2D layered-structure with a 6-fold coordination of Mg cations up to the highest pressure of this study. The results are discussed within the context of the well established structural behavior of highly compressed AX_2_ compounds.

## Results

### Structural properties under pressure

In Fig. 2 we present integrated diffraction patterns of MgCl_2_ at selected pressures up to 100 GPa. The evolution of the XRD data shows discontinuous changes beginning at approximately 0.7 GPa thus revealing the occurrence of a phase transition. Above 50 GPa, Bragg peak intensity decreases and peak widths increase, which may signal the onset of amorphization; however, the most intense high pressure phase (HP) Bragg peaks can be followed up to 100 GPa without any sign of a subsequent phase transition. The HP phase Bragg peaks can be systematically indexed with the hexagonal CdI_2_-type structure. To determine the structural parameters the diffraction patterns were analyzed by performing Rietveld refinements using the GSAS^23^ software. In both structures, the only free positional parameter is the z-coordinate of Cl^−^ anions. In detail, Mg atoms occupy the 3a(0, 0, 0) and 1a(0, 0, 0,) while Cl atoms the 6c(0, 0, z) and 2d(1/3, 2/3, z) Wyckoff Positions (WP) in ambient and HP phases respectively. Examples of refined profiles are plotted in the Supplementary information Fig. 1.

**Figure 1:**
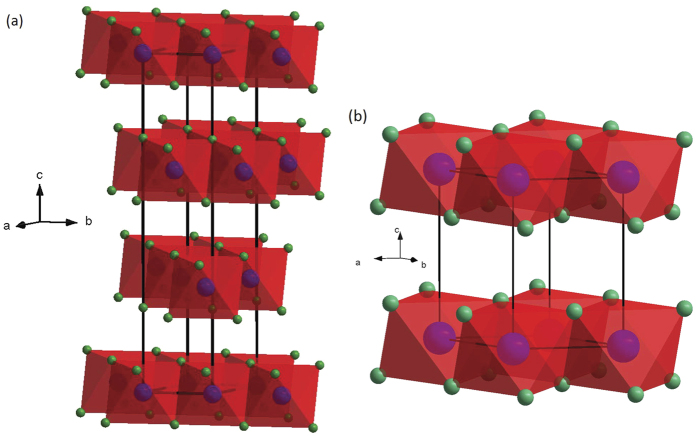
Schematic representations of: (**a**) CdCl_2_-type and (**b**) CdI_2_-type crystal structures of MgCl_2_. Blue and green spheres indicate Mg and Cl anions respectively.

**Figure 2:**
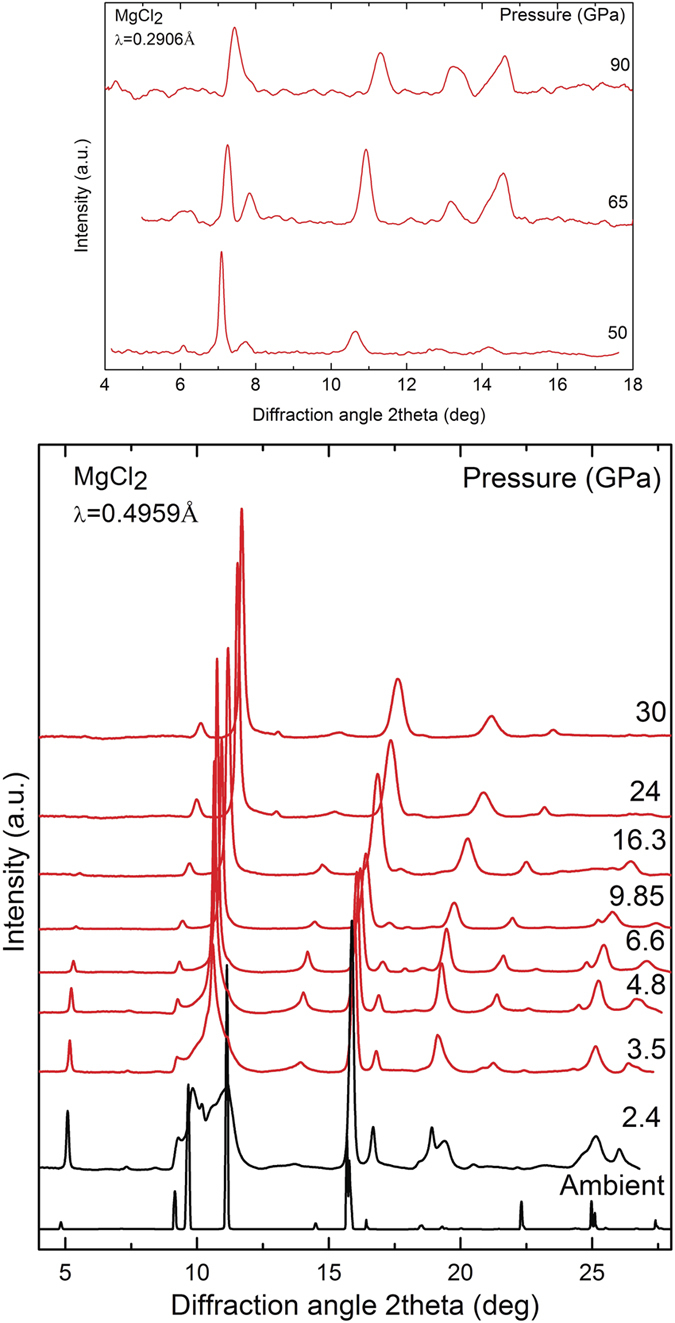
XRD patterns of MgCl_2_ at selected pressures. The patterns at 2.4 and 3.5 GPa correspond to a phase mixture of CdCl_2_- and CdI_2_-type phases.

**Figure 3:**
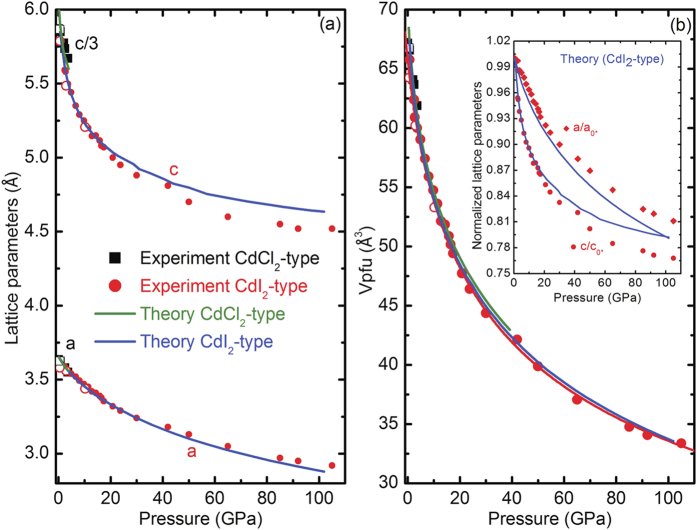
(**a**) Pressure dependence of the lattice parameters and (**b**) volume-pressure data for CdCl_2_- and CdI_2_-type phases of MgCl_2_. The solid red line is a third-order BM equation of state fit of the HP phase experimental data. The inset in (**b**) shows the normalized lattice parameters of the CdI_2_-type phase. The asterisks denote the values of the lattice parameters at the onset pressure of the HP phase. Experimental results are represented by solid and open symbols during compression and decompression respectively. Calculated values are represented by solid green and blue lines for CdCl_2_- and CdI_2_-type phases respectively.

**Figure 4:**
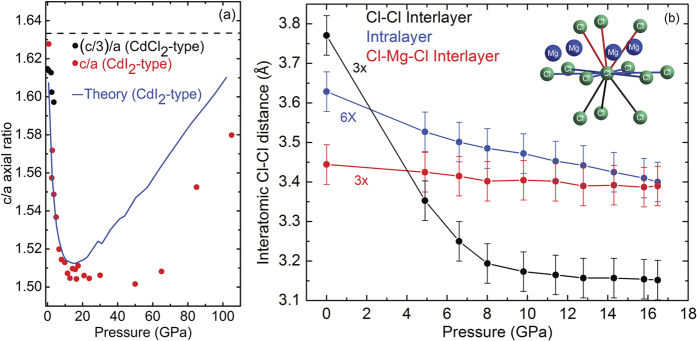
(**a**) The pressure dependent c/a axial ratio of MgCl_2_. The horizontal dashed black line represents the ideal hexagonal c/a ratio value. Experimental results are represented by solid symbols and the calculated values are represented by a solid blue line. (**b**) Three different pressure dependent Cl-Cl distances: (i) interlayer between Cl anions of different Cl-Mg-Cl “sandwich” (black), (ii) intralayer Cl-Cl (blue), and (iii) internal Cl anions within the same Cl-Mg-Cl “sandwich” (red).

We determined pressure dependent lattice parameters, cell volumes per formula unit (V_*p*.*f*.*u*._), and interatomic distances for both MgCl_2_ structures. Experimental and theoretical results are plotted in Fig. 3. The experimental and theoretical lattice parameters and the (V_*p*.*f*.*u*._) values match quite well for both phases. The maximum difference between calculated and measured c- and a- values is less than 2.9% and 1.0% respectively (Fig. 3a). These results increase confidence in the accuracy of the theoretical methods used in this work. The corresponding structural details are summarized in Table 1. The observed continuity of lattice parameter values (with *c*_*HP*_ = *c*_*amb*_/3) and V_*p*.*f*.*u*._ before and after the phase transition signals a second-order phase transition much like the case of FeCl_2_^10^. However, the two-phase coexistence pressure range of ≈3 GPa appears to be much more extensive than in the case of FeCl_2_. The compressibility of the HP phase c-axis (Fig. 3(b) inset) exceeds the a-axis during initial compression up to 10 GPa thus reflecting, as normally expected due to the weak Van der Waals interlayer forces, higher compressibility in the direction perpendicular to the layers that reduces the free interlayer spacing (Fig. 1). This phenomenon is also reflected by the decreasing c/a axial ratio, which abruptly becomes pressure invariant at 10 GPa (Fig. 4(a)). We note that the initial c/a axial ratio is very close to the ideal hexagonal ratio 1.633, and it strongly decreases with increasing pressure until reaching an effectively pressure independent value of ≈1.51 above 10 GPa and up to 50 GPa. A similar trend is reported in the case of FeCl_2_^11^. However, in the present study the c/a ratio remains constant in this pressure range; there is no indication of an abrupt change of lattice parameters or discontinuous change of the pressure dependence of the axial ratio^11^. This can be attributed to the absence of an electronic phase transition in MgCl_2_ as opposed to FeCl_2_, *i.e.* in this study we observe the *pure* physical effect of compression on the crystal structure, that is, pressure homogenizes the structure. The constant value (~1.51) of c/a ratio above 10 GPa reflects the reduced compressibility of the c-axis, which becomes equal to the a-axis at least below 60 GPa in the experimental data (Fig. 3(b) inset). The origin of this effect can be understood in terms of the three different pressure dependent Cl-Cl interatomic distances plotted in Fig. 4(b). Initial compression mainly affects the interlayer Cl-Cl distance while Cl-Cl distances within individual Cl-Mg-Cl “sandwich” are only slightly affected. At 10 GPa, the interlayer Cl-Cl distance becomes shorter than the internal Cl-Mg-Cl “sandwich” Cl-Cl separation; here it is plausible to assume that the increased charge repulsion between interlayer Cl-Cl anions serves to markedly reduce the c-axis compressibility. We also note that the data plotted in Fig. 4 stops at 16 GPa. Although initial samples consisted of nearly perfect fine powdered grains yielding uniform intensity ring-like 2D XRD images, the phase transition increased the sample grain size distribution resulting in much less uniform (spotty) diffraction ring intensity profiles. Higher intensity large diffraction spots spatially broaden Bragg peaks. In addition to grain size induced broadened diffraction rings, pressure induced peak broadening also occurred thus preventing us from confidently determining Cl-Cl distances above 16 GPa. It is interesting that the c/a ratio increases above 20 GPa in the calculated and above 50 GPa in the experimental results. It is plausible that pressure dependent repulsion between the Cl^−^ anions reaches a threshold; a significant reduction in compressibility along the c-axis could be signaled by the inflected (increasing) c/a ratio.

Consistent with most high-pressure EOS studies, we conducted unweighted fits of the pressure-volume data to a third-order Birch-Murnaghan (B-M) equation of state^24^ and determined the bulk modulus *K*_*o*_ and its first derivative *K*′ at zero pressure for the CdCl_2_-type phase and at the experimental onset pressure for the CdI_2_-type phase. The elastic parameters obtained this way are given in Table 1 and are comparable with those reported for FeCl_2_^11^. The *K*_*o*_ values of both MgCl_2_ phases are about three times lower than the respective ones for the rutile-type fluoride counterparts, like MgF_2_ and MnF_2_^25^,^26^, although in both structures cations are 6-fold coordinated. This highlights the lower dimensionality-connectivity of the Lawrencite-type compounds in comparison to the 3D rutile-type compounds. Upon pressure release, the phase transition appears to be reversible with negligible hysteresis and MgCl_2_ returns back to the CdCl_2_-type structure.

**Figure 5:**
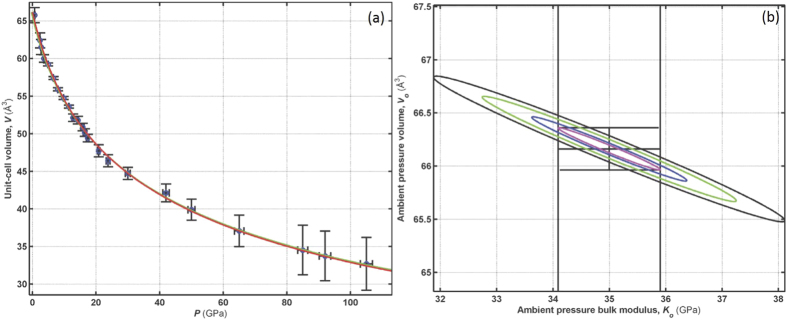
(**a**) MgCl_2_ high-pressure phase data fits, unweighted with green line and weighted with red line, using a 2nd order B-M EOS model. (**b**) The experimentally weighted V_0_ versus K_0_ confidence ellipses. The magenta colored ellipse is 0.607-*σ* (50.3% confidence), blue is 1-*σ* (68.3% confidence), green is 2-*σ* (95.4% confidence), and the black ellipse is 3-*σ* (99.7% confidence).

To gain deeper insight into how MgCl_2_ responds under quasi-static compression, we conducted weighted fits and used the reduced 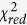 goodness-of-fit formalism to compare the effectiveness of three EOS models to represent the P-V data. The model that generates minimal parameter error and has a reduced 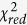 value closest to 1 represents the “ best-fit model”. We tested the Birch-Murnaghan^24^, (B-M), 2^*nd*^ to 5^*th*^ orders, the Vinet^27^, and the F-f 28 finite strain 1^*st*^ to 3^*rd*^ order EOS models for the ambient phase MgCl_2_. The first-order F-f model was sufficient to represent the data; however, the 2nd order B-M model is statistically better at representing the ambient phase MgCl_2_ data (see Table 2 and supplementary Figure 3). The same analytical approach was applied to the high-pressure phase except here we replace the F-f model with a corresponding linearized G-g stress-strain model where an arbitrary reference V_0_ value is chosen and the ambient pressure properties are then determined at *g* = *g*_*o*_ (strain at ambient pressure) using the G-g relation^29^ (see Supplementary Figure 4). Again the 2nd order B-M model was found to reasonably represent the data, see Table 2 and Fig. 5(a), although the G-g EOS model estimated standard deviation (esd) values are lower in magnitude. The computed G and g errors are relatively low and thus lead to more significantly weighted differences between the model and the measured pressure. This is why the G-g model 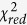 value is comparatively large despite the small maximum pressure difference from the data. The high pressure V_0_ value is just slightly smaller than the ambient pressure volume. What is perhaps unusual is that the low pressure phase is less compressible than the high pressure phase. If one takes into account the respective *K*_0_ esd’s, particulary in the case of the *α*-MgCl_2_, then the strength of this observation is not highly convincing. The relatively high *K*_0_ esd value for *α*-MgCl_2_ is a direct consequence of both the low P_*c*_ of the phase transition and the low number of the P-V data points for *α*-MgCl_2_. Moreover, the use of second-order B-M EOS models, K′ is fixed, further affects the K_0_ values. Refer to Supplementary Information for a description of the complete statistical analysis including fitting procedures. For the best-fit model, we plot corresponding two-dimensional confidence ellipses to reveal two-variable correlation information (Fig. 5(b)).

**Figure 6:**
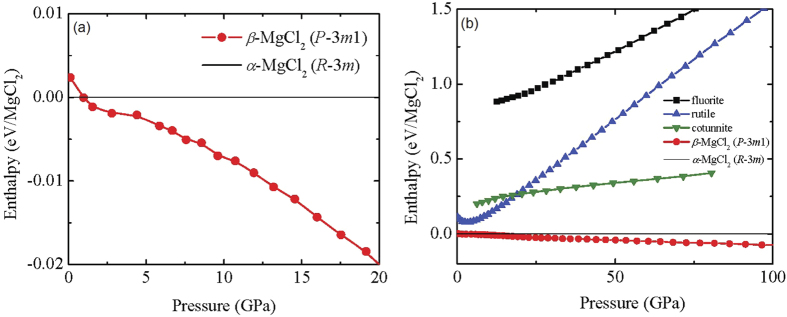
(**a**) Calculated pressure dependent enthalpy differences for *α*-MgCl_2_ and *β*-MgCl_2_ phases of MgCl_2_ up to 20 GPa. (**b**) Calculated enthalpy differences for *α*-MgCl_2_ and *β*-MgCl_2_ phases of MgCl_2_ together with the hypothetical rutile, fluorite and cotunnite phases as a function of pressure up to 100 GPa. The enthalpy of the *α*-MgCl_2_ phase is taken as the reference.

The calculated pressure dependent enthalpies for the *α*-MgCl_2_ and *β*-MgCl_2_ are shown in Fig. 6(a) over the pressure range 0–25 GPa. The calculation of the ambient pressure structure correctly predicts the *α*-MgCl_2_ polymorph as the thermodynamic ground state of MgCl_2_. The *β*-MgCl_2_ structure becomes more thermodynamically stable than the *α*-MgCl_2_ structure at ≈0.9 GPa, which agrees well with the experimentally measured transition pressure of 0.7 GPa.

### Raman scattering under pressure

**Figure 7:**
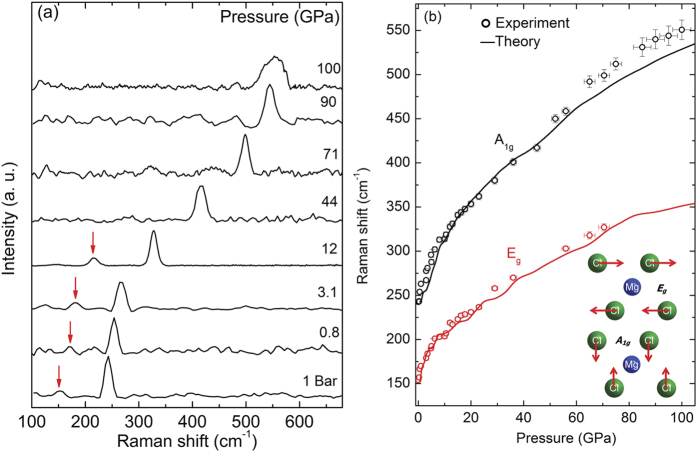
(**a**) Raman spectra of MgCl_2_ at various pressures. The low intensity *E*_*g*_ modes are marked with vertical red arrows. (**b**) Pressure dependent phonon frequencies. Experimental results are represented by open symbols and calculated values with solid lines, see supplementary information Fig. 2 for the low pressure data. The inset shows the eigenvectors of the Raman-active zone-center modes *A*_1*g*_ and *E*_*g*_.

Two Raman-active zone-center modes are predicted from group theory for both the *α*-MgCl_2_ and *β*-MgCl_2_ phases with the symmetries: *A*_1*g*_ + *E*_*g*_^30^. *A*_1*g*_ corresponds to the displacement of Cl along the c-axis and the *E*_*g*_ mode corresponds to lateral Cl displacements within the Cl-Mg-Cl layers^31^, see inset of Fig. 7(b). At ambient pressure, with MgCl_2_ encapsulated within the unpressurized DAC sample chamber, the observed Raman mode frequencies are in excellent agreement with previous studies^30^ (Fig. 7). The calculated frequencies for the *A*_1*g*_ and *E*_*g*_ modes are 245 cm^−1^ and 157 cm^−1^, respectively, which closely match with the experimental values of 242.6 cm^−1^ and 154 cm^−1^. Above 1 GPa, the number of observed Raman modes remains the same (Fig. 7(a)) and no apparent discontinuity of the Raman frequencies is observed (Fig. 7(b)). This observation is congruent with a second-order *α*-MgCl_2_ → *β*-MgCl_2_ phase transition and is moreover consistent with XRD measurements and relative enthalpy calculations. We calculated room-temperature phonon dispersion curves for *α*-MgCl_2_ at 0 and 0.67 GPa (see Supplementary information Fig. 3). Soft modes are clearly revealed at the T symmetry point, which is consistent with a second-order phase transition to the beta phase. Above 70 GPa, it is not possible to measure the low-frequency broad E_*g*_ mode. On the other hand, the *A*_1*g*_ mode is easily observed up to 100 GPa and its frequency varies smoothly with increased pressure (Fig. 7(b)). The continuity of the A_1*g*_ together with the absence of any new intense Raman peaks further justifies the argument that MgCl_2_ remains in the *β*-MgCl_2_ phase up to 100 GPa. The mode Grüneisen parameters (*γ*_*T*_) determined using the experimental results of this work are shown in Supplementary Table 2. The *γ*_*T*_ parameters of modes of the *β*-MgCl_2_ phase are common for materials with mixed ionic-covalent bonding (such as within the Cl-Mg-Cl layers)^32^, due to the presence of weaker interlayer bonds, which initially are more compressible.

## Discussion

As discussed in the introduction, we would normally expect MgCl_2_ to undergo a pressure-induced phase transitions towards higher connectivity (3D) and coordination number structures. According to the general systematics of pressurized AX_2_ compounds^33^ a typical *α*-quartz (rhombohedral S.G. *P3*_2_*21* (154) CN = 4) → rutile (tetragonal S.G. *P4*_2_*/mnm* (136) CN = 6) → CaF_2_-type (cubic fluorite SG *Fm-3m* (225) CN = 8) → *α*-PbCl_2_-type (cotunnite orthorhombic SG *Pnma* (62) CN = 9) → Ni_2_In (hexagonal S.G. *P6*_3_*/mmc* (194) CN = 11) sequence of phase transitions is expected^34^,^35^,^36^,^37^ with an overall increase in cation coordination number from 4 (*α*-quartz) to 11 (Ni_2_In). In this sequence, only the prototypical structures are noted because of the plethora of closely related alternative structural types. For instance one can group the CaCl_2_-type and the *α*-PbO_2_-type in the rutile family and the PdF_2_-type and FeS_2_-type (pyrite) in the fluorite family, see discussion in ref. 26 and Fig. 1 of ref. 38. So, the application of pressure results in higher coordination number (4 to 11) and phase transitions resulting in structures with characteristically higher R = *r*_*c*_/*r*_*a*_ values due to the well known higher compressibility of anions in comparison to cations^39^. Here we summarize the pressure induced phase transitions of few AX_2_ systems relevant to MgCl_2_, *i.e.* with low R = *r*_*c*_/*r*_*a*_ values at ambient conditions. SiO_2_, which is considered as the AB_2_ compound with the highest pressure phase transition, crystallizes in the *α*-quartz structure at ambient conditions and transforms to rutile (Stishovite) above 10 GPa^40^. MgF_2_ crystallizes to the rutile structure at ambient conditions and transforms to a modified fluorite structure (PdF_2_-type) above 14 GPa and to the cotunnite structure above 35 GPa. Although there are no experimental data on BeX_2_ compounds a very recent theoretical study of BeF_2_^41^ suggests that the ambient pressure *α*-quartz structure transforms to rutile above 27 GPa. Finally, CaCl_2_ crystallizes in the CaCl_2_-type (rutile family) at ambient pressure and transforms to cotunnite above 10 GPa.

As evident from our XRD and Raman spectroscopy results, MgCl_2_ remains in the *β*-MgCl_2_ 2D layered structure up to 100 GPa A kinetic barrier could be inhibiting the expected phase transformation. This phenomenon has been observed in the case of the SiO_2_ for the *α*-PbO_2_-type phase transitioning to the PdF_2_-type phase above 268 GPa^42^ and also in the cases of CaF_2_ and SrF_2_^43^ transitioning from the cotunnite-type phase to the Ni_2_In-type structure. In the aforementioned examples higher temperatures, using laser heating, were needed to overcome the kinetic barriers. In order to elucidate if the same scenario applies to MgCl_2_ we performed pressure dependent first-principles enthalpy calculations for the *β*-MgCl_2_ phase and the hypothetical rutile, fluorite and cotunnite phases of MgCl_2_. The results are plotted in Fig. 6(b). As it can be clearly seen from the enthalpy vs pressure plot, *β*-MgCl_2_ remains the more stable phase up to 100 GPa. Moreover, the enthalpy difference between the *β*-MgCl_2_ and the candidate rutile, fluorite and cotunnite phases increases with pressure. Thus, it is not expected that these structures will become energetically favorable even above 100 GPa. Although slight modifications of the prototypical candidate structures might have lower enthalpies than the prototypical ones it is expected that the difference between them should be much lower than the difference with *β*-MgCl_2_. Moreover, it is normally expected that the enthalpy difference between the *β*-MgCl_2_ and these slight modifications will also increase with pressure^26^,^38^,^44^.

From the above discussion it is clear that MgCl_2_ is a rare exception; it does not follow the general structural trend of highly compressed AX_2_ compounds up to the maximum pressure of this study. One possible scenario is that in order for Mg to have an increased coordination number a precondition must first be met, what we may call, for the sake of brevity, layer mixing, which is inhibited by charge repulsion between interlayer Cl-Cl anions. However, this scenario implies that a kinetic barrier is at play, the existence of which is not supported by the results of our first-principles enthalpy calculations. It does appear that once a layered structure is established (*α*-MgCl_2_ and *β*-MgCl_2_ have similar structural characteristics and enthalpies) with anions positioned in an ideal close-packed arrangement, as opposed to buckled hcp layers in rutile, then the enthalpy of the system reaches a profoundly deep minimum well. A closer look at the hypothetical rutile-type structure of MgCl_2_ reveals that the MgCl_6_ octahedron in this structure is axially distorted. Specifically, two Mg-Cl bonds in the MgCl_6_ octahedron are compressed along the axial direction, forming a D_4*h*_ point group that is similar to the coordination of high-spin manganese (III). For example, at 100 GPa, the two shorter Mg-Cl bonds are 1.84 Å and the four longer ones are 1.907 Å, respectively, in the rutile structure. Such a distortion is energetically unfavorable. On the other hand, in the *R-3m* and *P-3m1* structures the MgCl_6_ octahedra maintain the O_*h*_ symmetry even at the Mbar region, thus these two structures are energetically more favorable than the rutile structure. The axial compression of the MgCl_6_ octahedron in the rutile structure is opposite to what one would expect from the Jahn-Teller stabilization, which is another interesting topic but clearly beyond the current scope of this paper. Electronic transitions do seem to affect structural stability or instability. In the case of the sister compound FeCl_2_, a second-order phase transition from the ambient CdCl_2_-type phase to the CdI_2_-type has been observed at very low pressure (ca 0.6 GPa)^10^. This phase remains stable without any sign of a structural phase transition, although two electronic phase transitions were reported, up to 65 GPa^11^, *i.e.*, FeCl_2_ remained in a layered motif up to this pressure. The high energy of the 3*d* orbital level in Mg, prohibits the *s* to *d* electron transition to occur in MgCl_2_ in the Mbar regions. Such electron transition would otherwise induce transition-metal behaviors for the metal and lead to new structures, for example, those observed in CaCl_2_ above 10 GPa. Pressure induced structural phase transitions, to yet unidentified structures, have been reported in the case of ZrS_2_ (at 8 GPa) and PbI_2_ (at 0.9 and 5 GPa)^45^, both have the CdCl_2_-type structure at ambient pressure. To the best of our knowledge, these are the only cases of reported pressure induced structural phase transitions of CdCl_2_-type compounds.

The equation of state of MgCl_2_, determined here from experimental data, is used to constrain thermochemical equilibrium calculations^46^ for Mg-containing energetic formulations that likely produce Mg, MgO, Mg(OH)_2_, and MgCO_3_ products. The addition of relevant EOS product species data improves the confidence of semi-empirical calculations of extreme condition thermochemistry. Chemical formulations can be *in silico* tuned to optimize conditions and products required to more efficiently neutralize biological threats. The EOS data determined in the present study will, in part, enable the development of thermochemical prediction tools to guide the development of efficient bio-agent defeat energetic formulations by optimizing the production of chosen specific biocidal products at detonation conditions.

In conclusion, the quasi-hydrostatic high-pressure structural dynamics of *α*-MgCl_2_ have been characterized by a combined experimental and first-principles study up to 100 GPa. A second-order phase transition to the *β*-MgCl_2_ phase has been observed to occur at approximately 0.7 GPa. We report a complete pressure dependent structural analysis including a systematic determination of pressure dependent Cl-Cl interatomic distances, which provides a clear understanding of the observed anisotropic compliance along the c-axis during initial compression and after its abrupt pressure invariance occurs above 10 GPa. Our results reveal that *β*-MgCl_2_ is unexpectedly stable up to the highest pressure of this study. The ideally positioned closed-packed Cl anion arrangement and lack of *interfering* electronic transitions can be partially attributed to the robustness of the *β*-MgCl_2_ hexagonal primitive cell structure. These results provide valuable insight into the high-pressure response and surprising stability of 2D layered CdI_2_-type compounds.

## Methods

### Experimental study

High purity (>99.99%) commercially available (Sigma-Aldrich) MgCl_2_ was ground to fine powder for x-ray diffraction (XRD) measurements. The sample including pressure sensors were loaded into diamond-anvil cell (DAC) sample chambers. For each of two x-ray studies, rhenium gaskets (preindented to 40–45 *μ*m thick using 400 *μ*m or 100 *μ*m diameter beveled culets) were used to radially confine the pressurized samples. Initial sample chamber diameters were nominally 150 *μ*m or 30 *μ*m for the smaller culet. Silicone oil was utilized as a pressure-transmitting medium (PTM) for XRD at low pressures and Ne for XRD at higher pressures including Raman measurements. Pressure was determined using a known ambient temperature EOS of gold^47^ and also using a calibrated ruby luminescence scale^48^. Integration of powder diffraction images to yield scattering intensity versus 2*θ* patterns and initial analysis were performed using the DIOPTAS^49^ program.

Image plate CCD detectors were used to collect pressure dependent X-ray diffraction data at the Advanced Light Source Beamline 12.2.2. An X-ray wavelength of *λ* = 0.4959Å was selected using a Si(111) double-crystal monochromator. Exposures time varied between 10 and 30 secs. The sample to detector distance of 300 mm was determined using a CeO_2_ (or LaB_6_) diffraction pattern. The X-ray beam was focused to 10 × 10 *μ*m using Kirkpatrick-Baez mirrors. More details on the experimental set up are given in Kunz *et al*.^50^. XRD data at pressures above 30 GPa were collected at the Extreme Conditions Beamline P02.2 at DESY (Germany)using a PerkinElmer detector^51^. The monochromatic x-ray beam (wavelength *λ* = 0.2898 Å) was focused to a nominal diameter of 4*μ*m using Kirkpatrick-Baez mirrors.

### First-Principles Theoretical Calculations

To determine the equation of state of MgCl_2_, we performed density functional calculations of pressure dependent enthalpies for a number of different crystal structures including the experimentally determined *α*-MgCl_2_ and *β*-MgCl_2_ phases. The additional calculated structures include fluorite (*Fm-3m*), rutile (*P4*_2_*/mnm*), and cotunnite (*Pnma*). The Vienna *ab initio* Simulation Package (VASP) program^52^ was used for total energy and lattice dynamics calculations combined with the projected augmented wave (PAW) potential^53^,^54^, the Perdew-Burke-Ernzerhof (PBE) exchange-correlation functional^55^ and a kinetic energy cutoff of 500 eV. The 2p^6^3s^2^ for Mg and 3s^2^3p^5^ for Cl were treated as valence states. Dense k-point grids^56^ were employed to sample the first Brillouin zone (BZ) for candidate structures, which yielded energies that converged to within 1 meV/atom. Specifically, the k-point grids used in the total-energy calculations are, 12 × 12 × 12 for the *α*-MgCl_2_, 12 × 12 × 8 for the *β*-MgCl_2_, 12 × 12 × 12 for the fluorite structure, 8 × 8 × 12 for the rutile structure, and 8 × 12 × 6 for the cotunnite structure, respectively. Lattice dynamics calculations were performed employing the density functional perturbation method, where the Hessian matrix and the vibrational frequencies were determined at the BZ center. A 12 × 12 × 12 and 12 × 12 × 8 k-point mesh was used for *α*-MgCl_2_ and *β*-MgCl_2_, respectively in these lattice dynamics calculations. Room temperature phonon dispersion relations were calculated employing the self-consistent *ab initio* lattice dynamical (SCAILD) method^57^ through a combination of VASP^52^ and PHON^58^ programs. SCAILD calculations were carried out employing supercells of 81 atoms for both *α* and *β* phases to yield the phonon frequencies that are converged within 0.05 THz (1.67 cm^−1^).

### Thermochemical calculations

Chemical equilibrium thermochemical calculations, based on a statistical mechanics theory of multi-component multi-phase reactive mixtures parameterized by experimental and simulation data^46^,^59^ typically yields thermodynamic predictions at extreme conditions that differ by less than 1–2% from the experimental results for a wide range of energetic materials.

## Additional Information

**How to cite this article**: Stavrou, E. *et al*. High-pressure X-ray diffraction, Raman, and computational studies of MgCl_2_ up to 1 Mbar: Extensive pressure stability of the *β*-MgCl_2_ layered structure. *Sci. Rep.* **6**, 30631; doi: 10.1038/srep30631 (2016).

## Supplementary Material

Supplementary Information

## Figures and Tables

**Table 1:** Experimental and calculated structural parameters of CdCl_2_- and CdI_2_-type phases of MgCl_2_ at selected pressures.

P(GPa)	SG	Z	*a*(Å)	*c*(Å)	*V*_*pfu*_(Å^3^)	*Ko*(GPa)	*K*′	WP	x	y	z
0	*R-3m*	3	3.635(1)	17.608(3)	67.16(2)	38.5(1.6)	4(fixed)	Cl(6c)	0	0	0.2557(2)
0 (calc.)	*R-3m*	3	3.651	17.785	68.44	22.7(0.4)	5.6(0.1)	Cl(6c)	0	0	0.2574
0^13^		3	3.6263	17.6663	67.43			Cl(6c)	0	0	0.2578
9.8	*P-3m1*	1	3.472(5)	5.212(4)	54.33(7)	32.3(1.9)	4.3(0.3)	Cl(2d)	1/3	2/3	0.264(3)
9.8 (calc.)	*P-3m1*	1	3.4567	5.2388	54.211	27.6(0.5)	4.7(0.1)	Cl(2d)	1/3	2/3	0.2604
10.1^11^	FeCl2	1	3.4188	5.221	52.85	35.3(1.8)	4(fixed)	Cl(2d)	1/3	2/3	0.270

Listed parameters include space group (SG), number of formula units in the unit cell Z, lattice parameters, cell volume per formula unit, bulk modulus *K*_*o*_ and the pressure derivative *K*′ as determined by unweighted least square fits, and the Wyckoff site with corresponding coordinates. Theoretical values (calc.) are listed below experimental values. Corresponding values from ref. 13 for MgCl_2_ at ambient pressure and from ref. 11 for FeCl_2_ at 10.1 GPa are provided for comparison.

**Table 2:** Parameters of the most optimal EOS model derived from fits to our MgCl_2_ data weighted according to experimental uncertainties.

*α*-MgCl_2_ phase											
B-M order	V_0_(Å^3^)	V_0_ esd (Å^3^)	K_0_ (GPa)	K_0_ esd (GPa)	K′	K′ esd	K″	K″ esd	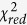	Max ΔP (GPa)	KS-test
2	67.1633	0.0003	46.0715	9.0364	4	0	[−0.0844]	[0.0166]	0.54	0.83	0.3
*β*-MgCl_2_ phase											
B-M order	V_0_(Å^3^)	V_0_ esd (Å^3^)	K_0_ (GPa)	K_0_ esd (GPa)	K′	K′ esd	K″	K″ esd	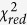	Max ΔP (GPa)	KS-test
2	66.1617	0.2360	34.9964	1.0735	4	0	[−0.1111]	[0.0034]	0.48	1.5	0.21
G-g order	V_0_(Å^3^)	V_0_ esd (Å^3^)	K_0_ (GPa)	K_0_ esd (GPa)	K′	K′ esd	K″	K″ esd	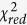	Max ΔP (GPa)	KS-test
1	65.9188	0.4917	34.1212	0.2206	4	0	[−0.1140]	[0.0065]	8.23	0.10	0.25

Note: *K*″ (bracketed terms) is implied (See: O.L. Anderson, 1995 Oxford Univ. Press^60^).
