# Supplementary material for: High-pressure X-ray diffraction, Raman, and computational studies of MgCl2 up to 1 Mbar: Extensive pressure stability of the β-MgCl2 layered structure
Source: Sci Rep. 2016 Aug 12;6:30631. doi: 10.1038/srep30631 (PMC4981878; doi:10.1038/srep30631)
Supplement: Supplementary Information [file srep30631-s1.pdf]

Supplementary Information

High-pressure X-ray diffraction, Raman, and computational studies of $MgCl_2$ up to 1 Mbar: Extensive pressure stability of the -$MgCl_2$ layered structure.

Elissaios Stavrou,[1,*] Yansun Yao,[2,3,†] Joseph M. Zaug,[1] Sorin Bastea,[1] Bora Kalkan,[4,5] Zuzana Konôpková,[6] and Martin Kunz[4]

[1]*Lawrence Livermore National Laboratory, Physical and Life Sciences Directorate, P.O. Box 808 L-350, Livermore, California 94550, USA*

[2]*Department of Physics and Engineering Physics, University of Saskatchewan, Saskatoon Saskatchewan, S7N 5E2, Canada*

[3]*Canadian Light Source, Saskatoon, Saskatchewan, S7N 2V3, Canada*

[4]*Advanced Light Source, Lawrence Berkeley Laboratory, Berkeley, California 94720, United States*

[5]*Advanced Materials Research Laboratory, Department of Physics Engineering, Hacettepe University 06800, Beytepe, Ankara, Turkey*

[6]*DESY Photon Science, D-22607 Hamburg, Germany*

(Dated: July 29, 2016)

* E-mail E.S. stavrou1@llnl.gov

† E-mail Y. Y yansun.yao@usask.ca

Contents:

- Reitveld refinements: Figure S1
- Lattice parameters and Volume$_{p.f.u.}$ as a function of pressure: Table S1
- Results of the analysis of Raman data: Table S2
- Raman peaks frequencies plotted against pressure up to 5 GPa: Figure S2
- Calculated room-temperature phonon dispersion curves for the α-$MgCl_2$ at 0 and 0.67 GPa: Figure S3
- Complete EOS analysis and evaluation procedure of three different models: Table S3 and Figures S4,5

COMPLETE EOS ANALYSIS AND EVALUATION PROCEDURE OF THREE DIFFERENT MODELS

In order to determine the EOS model(s) that best represent the experimental P-V data, we conducted weighted fits using Birch-Murnaghan, [1] (B-M), 2nd to 5th orders, the Vinet [2], and either the F-f [3] finite strain 1st to 3rd order for the ambient-phase or the linearized G-g stress-strain for the high-pressure phase EOS models. The fit results are summarized in Table I. The "best-fit model"was determined using simple statistical criteria: i) The uncertainties (errors) should be much lower than the corresponding fitting parameters; after applying this criteria all but the third-order B-M and Vinet models were excluded; ii) In the remaining two EOS models the reduced χ^2_{red} value closest to 1 criteria was applied. By doing so, the 3rd-order B-M model, instead of the Vinet EOS, was determined as the most optimal EOS model. The 3rd-order B-M model also yielded a more optimal KS-test value too where a value of zero is optimal. Lastly the 3rd-order B-M model had a lower maximum pressure difference with the data than the Vinet model. The third-order B-M model is clearly the "best-fit model".

The χ^2_{red} function is used with the assumption that measured values have uncorrelated Gaussian distributed error. For the case of a small number (N < 100) of data points, (like most high-pressure EOS studies), the uncertainty of χ^2_{red} values can be unacceptably large;

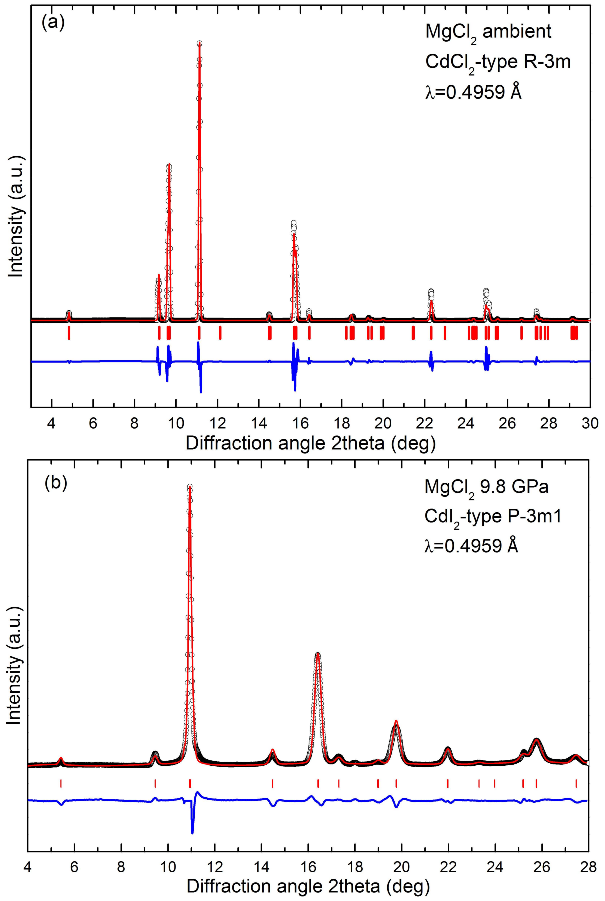

FIG. S1. Reitveld refinement results for $MgCl_2$ at: (a) ambient pressure,$CdCl_2$-type structure and (b) 9.8 GPa, CdI_2-type. Symbols correspond to the measured profile, the red solid lines represent the results of Rietveld refinements. The difference curves (blue curves) are shown also. Vertical tick marks indicate Bragg peak positions.

moreover, for nonlinear fitting forms such as higher order EOS models, the "hat"matrix does not exist. In other words, there is no reliable means to compute the number of degrees of freedom (NDF) for parameters in a nonlinear model; and further, NDFs can vary during an optimization search for a global minimum solution. For these reasons, we also conducted Kolmogorov-Smirnov tests [5, 6] (KS-test), *i.e.*, compared converged model fit residuals to a Gaussian distribution with a mean value μ= 0 and a variance of σ^2 =1. The bias (highest region of sensitivity) of a KS-test is selected by the comparative Gaussian mean value distribution value. In some reports, the KS-test has been proven to be more robust than the reduced χ^2_{red} formalism [7]. KS-test values range from 0 (optimal) to 1 (poor).

TABLE S1. Experimental lattice parameters and cell volume per formula unit of $CdCl_2$- and CdI_2-type phases of $MgCl_2$.

P(GPa)	a(Å)	c(Å)	V_{pfu}(Å^3)
	α-$MgCl_2$ phase		
0	3.635	17.608	67.16
0.7	3.625	17.552	66.57
2.0	3.584	17.320	64.08
2.4	3.582	17.211	63.67
3.6	3.554	17.013	61.88
	β-$MgCl_2$ phase		
0.7	3.621	5.863	66.57
2.4	3.591	5.591	62.44
2.71	3.557	5.584	61.18
3.6	3.552	5.498	60.07
4.9	3.540	5.442	59.06
6.6	3.526	5.350	57.60
8.2	3.493	5.293	55.93
9.8	3.473	5.254	54.88
11.4	3.455	5.205	53.80
12.8	3.422	5.146	52.18
14.3	3.413	5.148	51.93
15.8	3.389	5.115	50.87
16.5	3.377	5.079	50.16
17.4	3.355	5.074	49.46
20.8	3.323	5.050	48.29
23.8	3.297	4.952	46.62
30.2	3.241	4.882	44.36
42	3.183	4.812	42.22
50	3.132	4.745	40.31
65	3.053	4.645	37.49
85	2.971	4.557	34.83
92	2.952	4.523	34.13
105	2.928	4.505	33.45

TABLE S2. Vibrational mode assignments, ambient condition Raman frequencies ω_0 (in cm^{-1}), slopes $(\partial\omega/\partial P)_T$ (in cm^{-1} GPa^{-1}) and Grüneisen parameters γ_T=-$\partial(\ln\omega)/\partial(\ln V)|_T$ of the β-$MgCl_2$ phase.

Mode	ω_0	$(\partial\omega/\partial P)_T$	γ_T
E_g	160.8	6.3	1.05
A_{1g}	251.2	7.9	1.25

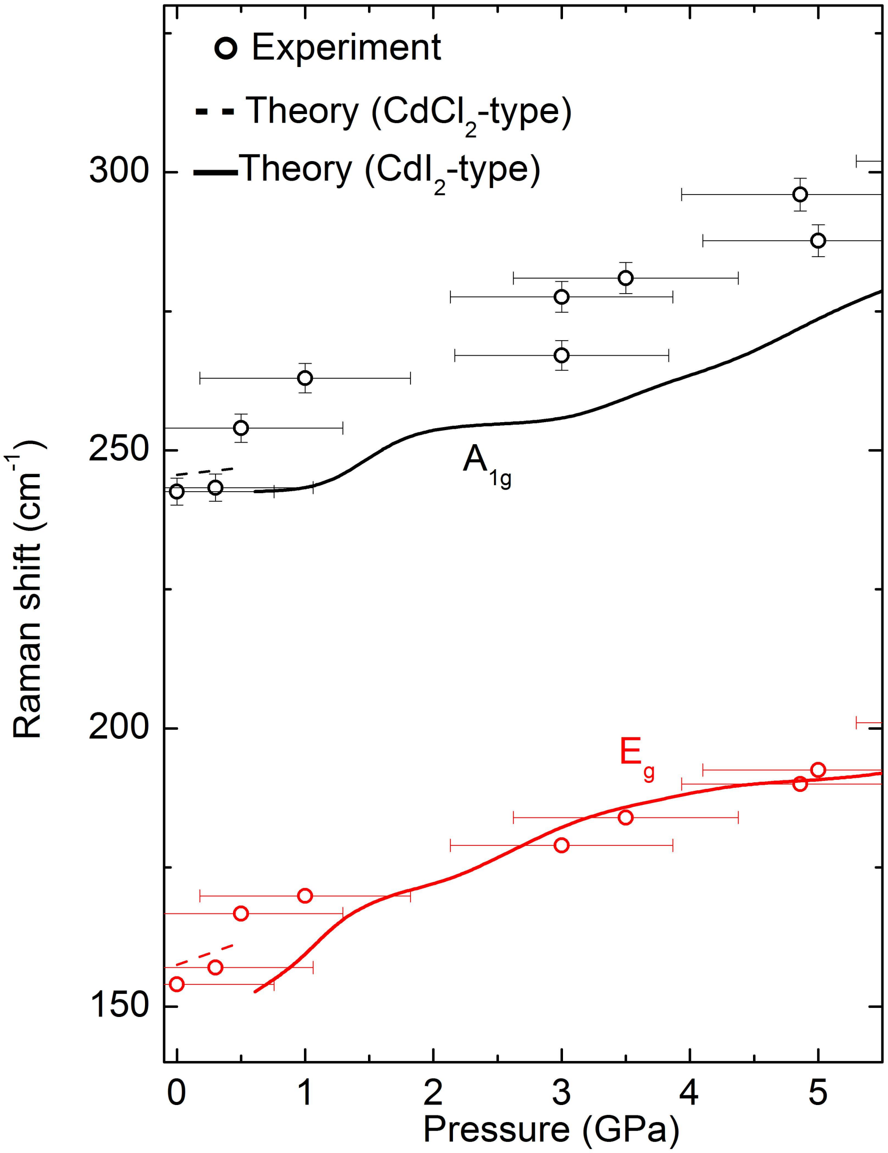

FIG. S2. Raman peaks frequencies plotted against pressure up to 5 GPa.

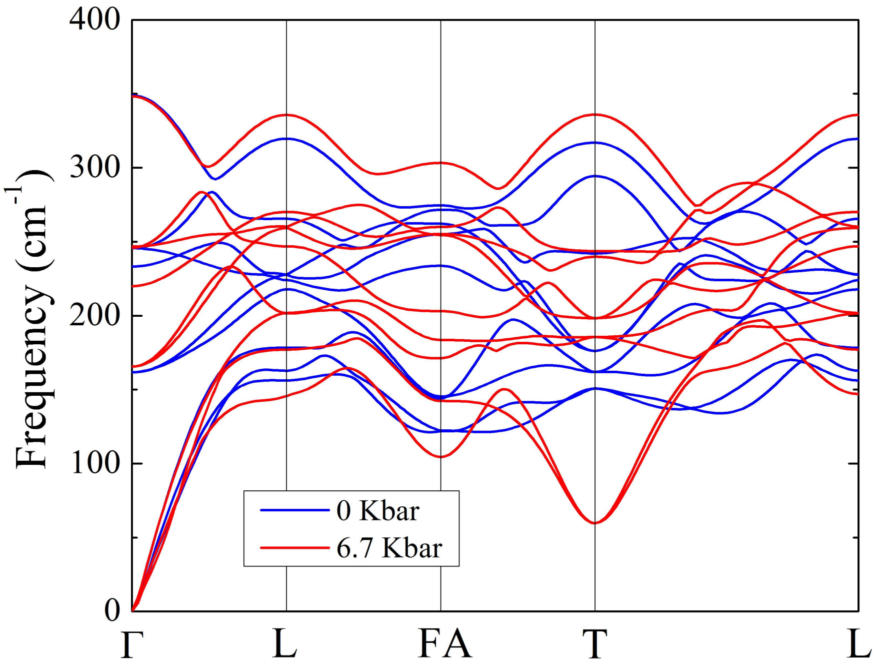

FIG. S3. Calculated room-temperature phonon dispersion curves for the α-$MgCl_2$ at 0 and 0.67 GPa.

TABLE S3. Model EOS parameters derived from fits to our $MgCl_2$ data, weighted according to experimental uncertainties. Note: *K"* (bracketed terms) is implied for 2nd and 3rd B-M and F(f) 1st order results (See: O.L. Anderson, 1995 Oxford Univ. Press [4]). The β-$MgCl_2$ phase fit results are based on fitting data for P < 30 GPa.

Experimentally Weighted Fits, α-$MgCl_2$ phase											
B-M order	V_0(Å^3)	V_0 esd	K_0(GPa)	K_0 esd	K'	K' esd	K"	K" esd	χ^2_{red}	Max ΔP (GPa)	KS-test
2	67.1633	0.0003	46.0715	9.0364	4.0000	0.0000	[-0.0844]	[0.0166]	0.54	0.83	0.3
Vinet EOS	V_0	V_0 esd	K_0	K_0 esd	K'	K' esd	K"	K" esd	χ^2_{red}	Max ΔP	KS-test
	67.1633	0.0003	47.5104	8.7785	-1.8278	4.6555	[0.0128]	[0.0406]	0.21	0.29	0.39
F-f order	V_0	V_0 esd	K_0	K_0 esd	K'	K' esd	K"	K" esd	χ^2_{red}	Max ΔP	KS-test
1	67.1619	0.0100	42.9589	2.7736	1.0043	1.4743	[-0.2297]	[0.1719]	0.14	36.65	0.48
Experimentally Weighted Fits, β-$MgCl_2$ phase											
B-M order	V_0(Å^3)	V_0 esd	K_0(GPa)	K_0 esd	K'	K' esd	K"	K" esd	χ^2_{red}	Max ΔP (GPa)	KS-test
2	66.1610	0.0108	30.9512	0.2035	4	0	[-0.1256]	[0.0008]	0.65	9.9	0.19
3	67.1624	0.0057	27.7109	0.6382	5.2407	0.2731	[-0.2407]	[0.0348]	0.63	29.0	0.33
Vinet EOS	V_0	V_0 esd	K_0	K_0 esd	K'	K' esd	K"	K" esd	χ^2_{red}	Max ΔP	KS-test
	67.1625	0.0057	27.3883	0.6182	5.5192	0.2610	[-0.3595]	[0.0321]	0.53	15.51	0.33
G-g order	V_0	V_0 esd	K_0	K_0 esd	K'	K' esd	K"	K" esd	χ^2_{red}	Max ΔP	KS-test
1	65.9187	0.5172	36.5513	0.1847	4	0	-0.1064	0.0051	6.6	0.24	0.25

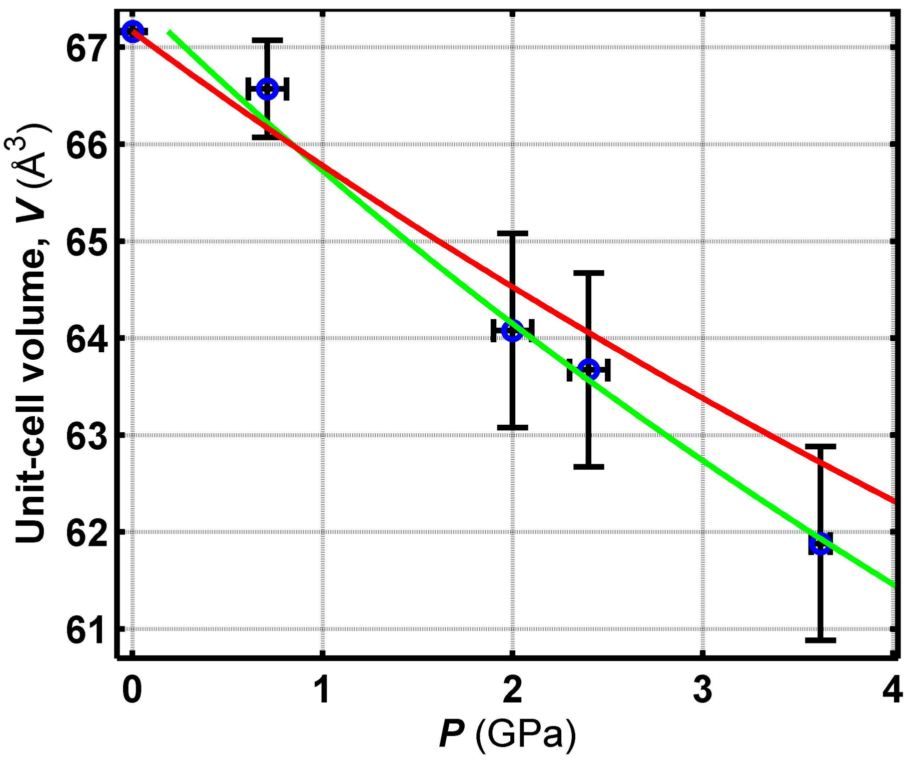

FIG. S4. $MgCl_2$ ambient pressure phase cold-compression data fit to a second-order B-M model. The green line represents an unweighted fit and the red line is an experimentally weighted fit.

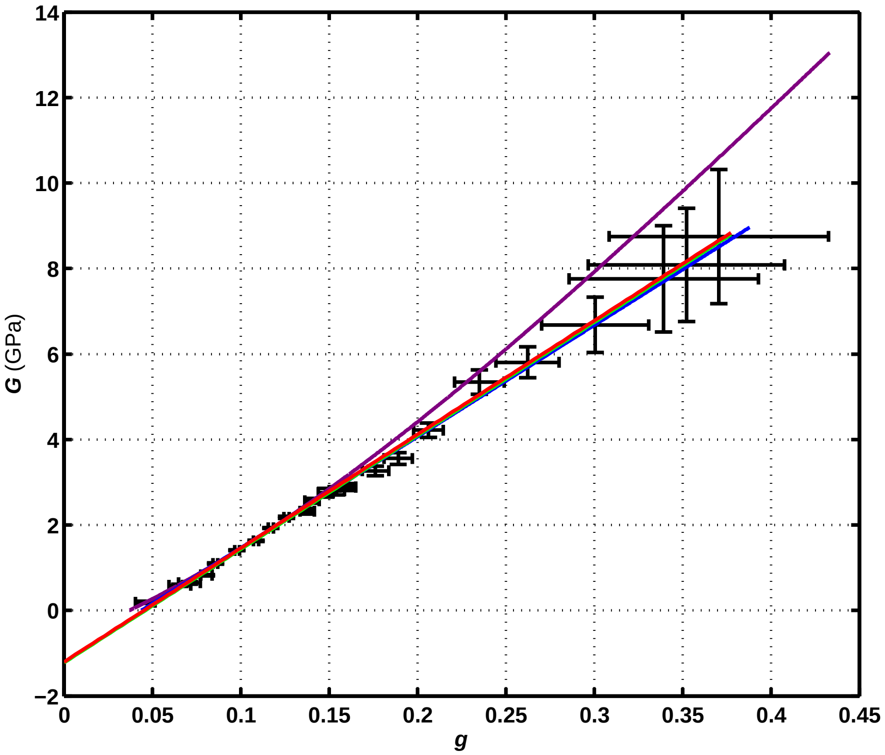

FIG. S5. $MgCl_2$ high pressure phase data fit using a 1st order G-g EOS model. The green line is an unweighted fit and the red line is an experimentally weighted fit. The blue line represents a 2nd-order B-M experimentally weighted fit and the violet line represents a Vinet EOS experimentally weighted model fit.

[1] Birch, F. Finite strain isotherm and velocities for single-crystal and polycrystalline nacl at high-pressures and 300k. *Journal Of Geophysical Research* **83**, 1257–1268 (1978).

[2] Vinet, P., Ferrante, J., Smith, J. R. & Rose, J. H. A universal equation of state for solids. *J. Phys. C* **19**, L467–L473 (1986).

[3] Birch, F. Finite elastic strain of cubic crystals. *Phys. Rev.* **71**, 809–824 (1947).

[4] Anderson, O. *Equations of State of Solids in Geophysics and Ceramic Science* (Oxford University Press Inc, 1995).

[5] Kolmogov, A. *Giornale dell Istitutano degli Attuari* **4**, 83 (1933).

[6] Smironov, N. *Annals of Mathematical Statistics* **19**, 279 (1944).

[7] Andrae, G., Schulze-Hartung, T. & Melchior, P. Arxiv e-prints 1012.3754v1 (2010).
